# CsCuAO1 Associated with CsAMADH1 Confers Drought Tolerance by Modulating GABA Levels in Tea Plants

**DOI:** 10.3390/ijms25020992

**Published:** 2024-01-12

**Authors:** Yu Cao, Yiwen Chen, Nuo Cheng, Kexin Zhang, Yu Duan, Shimao Fang, Qiang Shen, Xiaowei Yang, Wanping Fang, Xujun Zhu

**Affiliations:** 1 College of Horticulture, Nanjing Agricultural University, Nanjing 210095, China; 2020104087@stu.njau.edu.cn (Y.C.); 2021104084@stu.njau.edu.cn (Y.C.); 14221125@stu.njau.edu.cn (N.C.); 2019104086@njau.edu.cn (K.Z.); 2018204034@njau.edu.cn (Y.D.); fsm12340@163.com (S.F.); fangwp@njau.edu.cn (W.F.); 2 Tea Research Institute, Guizhou Provincial Academy of Agricultural Sciences, Guiyang 417100, China; shenqiang_gzu@163.com (Q.S.); yangxiaowei_gzu@163.com (X.Y.)

**Keywords:** tea plants, γ-aminobutyric acid, putrescine, drought tolerance, COPPER-CONTAINING AMINE OXIDASE, AMINOALDEHYDE DEHYDROGENASE

## Abstract

Our previous study showed that COPPER-CONTAINING AMINE OXIDASE (CuAO) and AMINOALDEHYDE DEHYDROGENASE (AMADH) could regulate the accumulation of γ-aminobutyric acid (GABA) in tea through the polyamine degradation pathway. However, their biological function in drought tolerance has not been determined. In this study, *Camellia sinensis* (*Cs*) CsCuAO1 associated with CsAMADH1 conferred drought tolerance, which modulated GABA levels in tea plants. The results showed that exogenous GABA spraying effectively alleviated the drought-induced physical damage. *Arabidopsis* lines overexpressing *CsCuAO1* and *CsAMADH1* exhibited enhanced resistance to drought, which promoted the synthesis of GABA and putrescine by stimulating reactive oxygen species’ scavenging capacity and stomatal movement. However, the suppression of *CsCuAO1* or *CsAMADH1* in tea plants resulted in increased sensitivity to drought treatment. Moreover, co-overexpressing plants increased GABA accumulation both in an *Agrobacterium*-mediated *Nicotiana benthamiana* transient assay and transgenic *Arabidopsis* plants. In addition, a GABA transporter gene, *CsGAT1*, was identified, whose expression was strongly correlated with GABA accumulation levels in different tissues under drought stress. Taken together, CsCuAO1 and CsAMADH1 were involved in the response to drought stress through a dynamic GABA-putrescine balance. Our data will contribute to the characterization of GABA’s biological functions in response to environmental stresses in plants.

## 1. Introduction

Drought is one of the major environmental stresses that affect plant growth and geographic distribution [1,2]. Tea plants (*Camellia sinensis*) frequently experience drought stress, usually caused by extreme high temperatures or irrigation limitation [3]. Drought is frequent and, because most tea cultivars are not drought-resistant, it causes significant losses to the tea industry all over the world [4]. Numerous studies have indicated that plants have developed various physiological, biochemical, and molecular mechanisms enabling them to adapt to a range of abiotic stresses [5]. Especially in field conditions, the effects of drought become more noticeable on tea plants; hence, improvements in drought resistance is necessary.

The γ-aminobutyric acid (GABA), a four-carbon non-protein amino acid, is generally accumulated under low-oxygen conditions (including waterlogging and hypoxia) [6]. The biological function of GABA has been thoroughly elaborated in animals, in which it acts as an inhibitory neurotransmitter, playing roles in reducing blood pressure, relieving insomnia, and alleviating depression. In plants, GABA accumulates quickly under various environmental stresses and also functions as a signal molecule [7]. Numerous studies have shown that the synthesis and accumulation of GABA improved tolerance and made the damage less severe in plants under drought stress [8,9]. In recent years, GABA-signal-induced stomatal closure in plants under drought stress has gained much attention [10,11]. The overproduction of reactive oxygen species (ROS) was reported to cause plant cell damage under drought stress, which can be reduced by GABA accumulation [6,9,11,12,13,14].

In higher plants, GABA is mainly produced from glutamate, catalyzed by glutamate decarboxylase (GAD) [15,16], some of which also comes from polyamine degradation [17,18]. The pathway of putrescine-derived GABA formation involves a two-step reaction, including diamine oxidase, COPPER-CONTAINING AMINE OXIDASE (CuAO), or polyamine oxidase catalytic processes and followed by AMINOALDEHYDE DEHYDROGENASE (AMADH) degradation [19,20].

The CuAOs convert primary amines to their aldehydes and release hydrogen peroxide (H_2_O_2_) [21] and play an important role in plant cell wall maturation and lignification, wound repair, and cell wall strengthening during pathogen infection [22,23]. Studies have shown that H_2_O_2_ produced by a CuAO catalytic reaction can regulate many development processes and defense responses, and it has also been reported that CuAO and its catalytic product H_2_O_2_ participated in abscisic-acid-induced stomatal closure [22,24]. The aldehyde dehydrogenase super-group is a family of enzymes that catalyze the NAD(P)+-dependent oxidation of endogenous and exogenous aldehydes to respective carboxylic acids [25], and plant AMADHs have generally been considered to perform substrate-dependent NADH production [26]. Although there is only one preliminary study of biochemical properties, AMADHs have become a hot topic in physiological processes [27]. Some other studies reported GABA accumulation in horticultural products, regulated by the AMADH function [15,28]. Previous studies showed that GABA was involved in a variety of abiotic and biotic stress responses, but very little is known about the involvement of CuAO1 together with AMADH1 in response to drought stress.

Our previous study reported that CuAOs and AMADHs could regulate GABA accumulation in tea plants through the polyamine degradation pathway [20]. Besides accumulation, we assessed what type of model could be used for drought tolerance in relation to GABA. Here, we demonstrated that exogenous GABA effectively alleviated drought-induced physical damage. The overexpression of *CsCuAO1* or *CsAMADH1* resulted in enhanced drought tolerance, which prompted ROS scavenging and stomatal movement, while the suppression of *CsCuAO1* or *CsAMADH1* could affect drought tolerance. Moreover, we also identified CsGAT1, a GABA transporter, whose expression was strongly correlated with GABA accumulation levels in different organs under drought stress. Our data suggested that CsCuAO1 and CsAMADH1 were involved in drought stress tolerance, which was regulated by a dynamic GABA–putrescine balance.

## 2. Results

### 2.1. Exogenous GABA Supply Enhances the Drought Tolerance of Tea Plants

**Figure 1:**
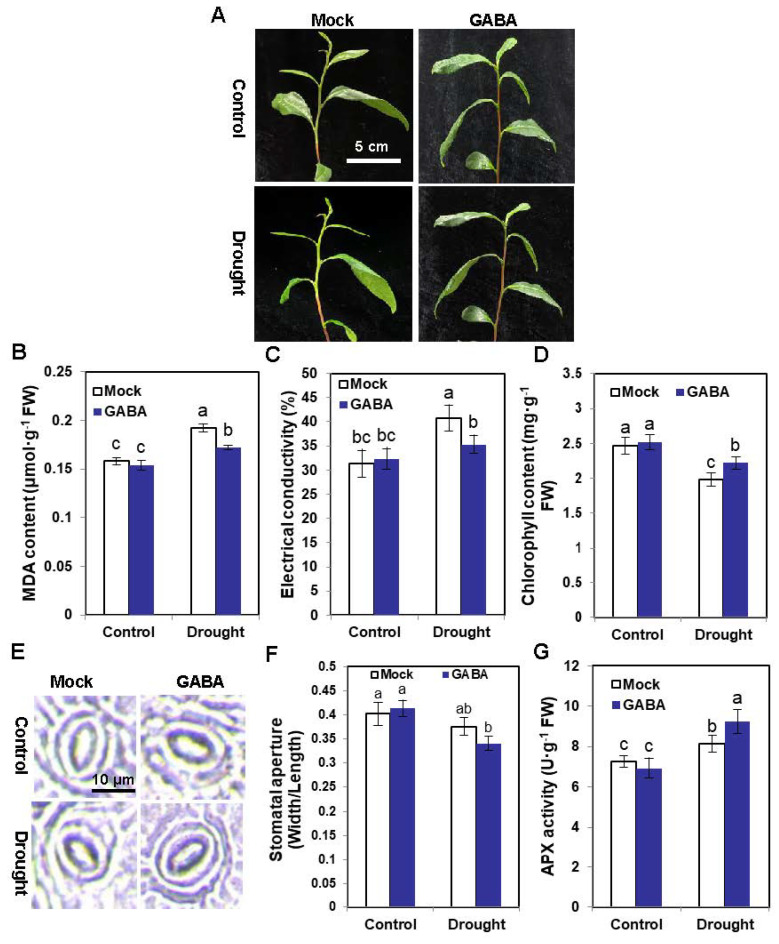
Exogenous GABA spraying enhances drought tolerance of tea plants. (**A**) Phenotypes of GABA-sprayed tea plants under control or 24 h of drought stress. Mock represents spraying with water. Scale bar = 5 cm. (**B**) The MDA content of GABA-sprayed tea plants under control or 24 h of drought stress. Mock represents spraying with water. (**C**) Electrical conductivity of GABA-sprayed tea plants under control or 24 h of drought stress. Mock represents spraying with water. (**D**) Chlorophyll content of GABA-sprayed tea plants under control or 24 h of drought stress. Mock represents spraying with water. (**E**) Stomatal images of guard cells of tea plant leaves under different treatments. Scale bar = 10 μm. (**F**) Stomatal apertures in guard cells of tea plant leaves under different treatments. At least 40 stomata were measured in each treatment. (**G**) The APX activity of GABA-sprayed tea plants under control or 24 h of drought stress. Mock represents spraying with water. Different lowercase letters over columns indicate significant differences between treatments (*p* < 0.05).

Tea plants sprayed with GABA suffered less damage after 24 h of drought treatment compared with the Mock tea plants (Figure 1A). Likewise, the malondialdehyde (MDA) content and electric conductivity of tea plants sprayed with GABA were both lower than those of the Mocks (Figure 1B,C). The chlorophyll content of tea plants sprayed with GABA was higher than that of the Mocks (Figure 1D). Microscope observations showed that the stomatal aperture of tea plants sprayed with GABA was narrower than that of the Mock plants under drought (Figure 1E,F). The ascorbate peroxidase (APX) activity of tea plants sprayed with GABA was higher than that of Mocks, further indicating that exogenous GABA could enhance the drought tolerance of tea plants (Figure 1G). Altogether, our data strongly suggested that exogenous GABA spraying enhanced drought tolerance in tea plants.

### 2.2. Drought Promotes the Transfer of GABA in Plants

**Figure 2:**
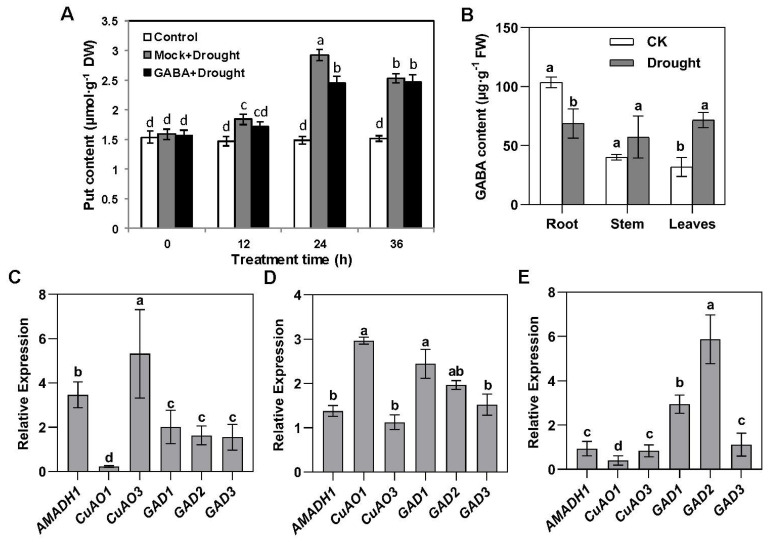
Putrescine and GABA accumulation levels, as well as expression profiles of putrescine-derived GABA-responsive genes in different tea organs under drought stress. (**A**) Quantitative analysis of putrescine in tea plant leaves with or without exogenous GABA spraying under drought stress at different times. Different lowercase letters over columns indicate significant differences between treatments (*p* < 0.05). Put, putrescine. (**B**) GABA concentration in different tea organs under control and drought stress. Different lowercase letters over columns indicate significant differences between treatments (*p* < 0.05). Fold change of expression of *CsGAD*s and putrescine-derived GABA-responsive genes (*CsAMADH1* and *CsCuAOs*) with drought treatment in tea plant (**C**) roots, (**D**) stems, and (**E**) leaves. Different lowercase letters over columns indicate significant differences between genes (*p* < 0.05).

Putrescine is an important precursor of GABA synthesis. After different time points of drought, the putrescine content in tea plants increased significantly. Interestingly, the putrescine content of plants sprayed with GABA was significantly lower than that of the Mocks at 12 and 24 h after drought treatment (Figure 2A). After drought treatment, the GABA content in roots decreased significantly and increased more than two times in leaves, but did not change in stems (Figure 2B).

The specific expression of putrescine-related genes in different parts of plants had attracted our attention. After drought treatment, the expression of *CsAMADH1* and *CsCuAO3* increased significantly in the tea roots (Figure 2C) and the expression of *CsAMADH1* and *CsCuAO1* increased significantly in the stems (Figure 2D). However, none of these increased in the leaves, and the expression of *CsCuAO1* even decreased (Figure 2E). The expression of *CsGAD1* and *CsGAD2* increased significantly in the stems and leaves but not in the roots (Figure 2C–E). Overall, our results suggested that the increased expression of *CsGAD*s under drought treatment led to an increased GABA content in stems and leaves. However, despite the upregulation of *CsAMADH1* and *CsCuAO3* in the roots, there was less GABA content in the roots. We speculated that there might be transporters coordinating the inter-tissue GABA content.

### 2.3. Identification and Characterization of CsGAT1

**Figure 3:**
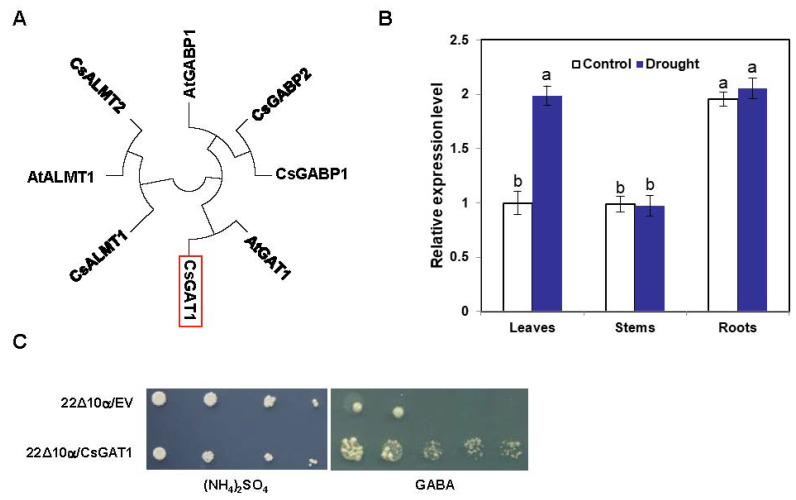
Identification and characterization of CsGAT1. (**A**) Phylogenetic analysis of CsGAT1 and other GABA transporters from plant species. (**B**) Relative expression level of *CsGAT1* in tea plants under normal and drought conditions. Different lowercase letters over columns indicate significant differences between treatments (*p* < 0.05). (**C**) Phenotypes of yeast strains with CsGAT recombinant plasmids grown on solid medium, with amino acid as the only nitrogen source.

To better understand how GABA in plants moved from the roots to stems and leaves under drought conditions, the GABA transporter gene had become the focus of our attention. AtGAT1 in Arabidopsis Thaliana has been confirmed to be a GABA transporter [29]. We searched the tea tree genome to find a gene with high homology to *AtGAD1*, named *CsGAT1* (Figure 3A). Under normal conditions, the expression of *CsGAT1* in the roots was significantly higher than in the leaves and stems. Interestingly, the drought treatment did not significantly alter the *CsGAT1* expression in roots or stems, but the expression in the leaves increased significantly (Figure 3B). Wild-type yeast can grow normally on a medium with amino acids as the only nitrogen source, but 22∆10α exhibits a growth deficiency phenotype due to its inability to properly absorb and transport amino acids (except arginine), so 22Δ10α was selected to explore whether CsGAT1 could transport GABA. On YNB medium with GABA as the sole amino acid source, the yeast mutant transformed with *CsGAT1* growing normally, while the yeast mutant transformed with the empty vector (EV); pYES2 did not grow (Figure 3C). The yeast mutant 22Δ10α expressing CsGAT1 was able to recover its growth defect phenotype. Therefore, we believe that CsGAT1 has GABA transport activity. When other amino acids were used as the sole nitrogen source, neither 22Δ10α/EV nor 22Δ10α/CsGAT1 grew normally (Figure S1). Therefore, combined with the changes in GABA content in the different plant tissues before and after drought treatment, we speculated that the GABA in the roots was transported to the leaves by CsGAT1 under drought treatment.

### 2.4. The Impact of Exogenous Putrescine on GABA Accumulation in CsAMADH1-, CsCuAO1-, and CsCuAO3-Overexpressing Arabidopsis Lines

**Figure 4:**
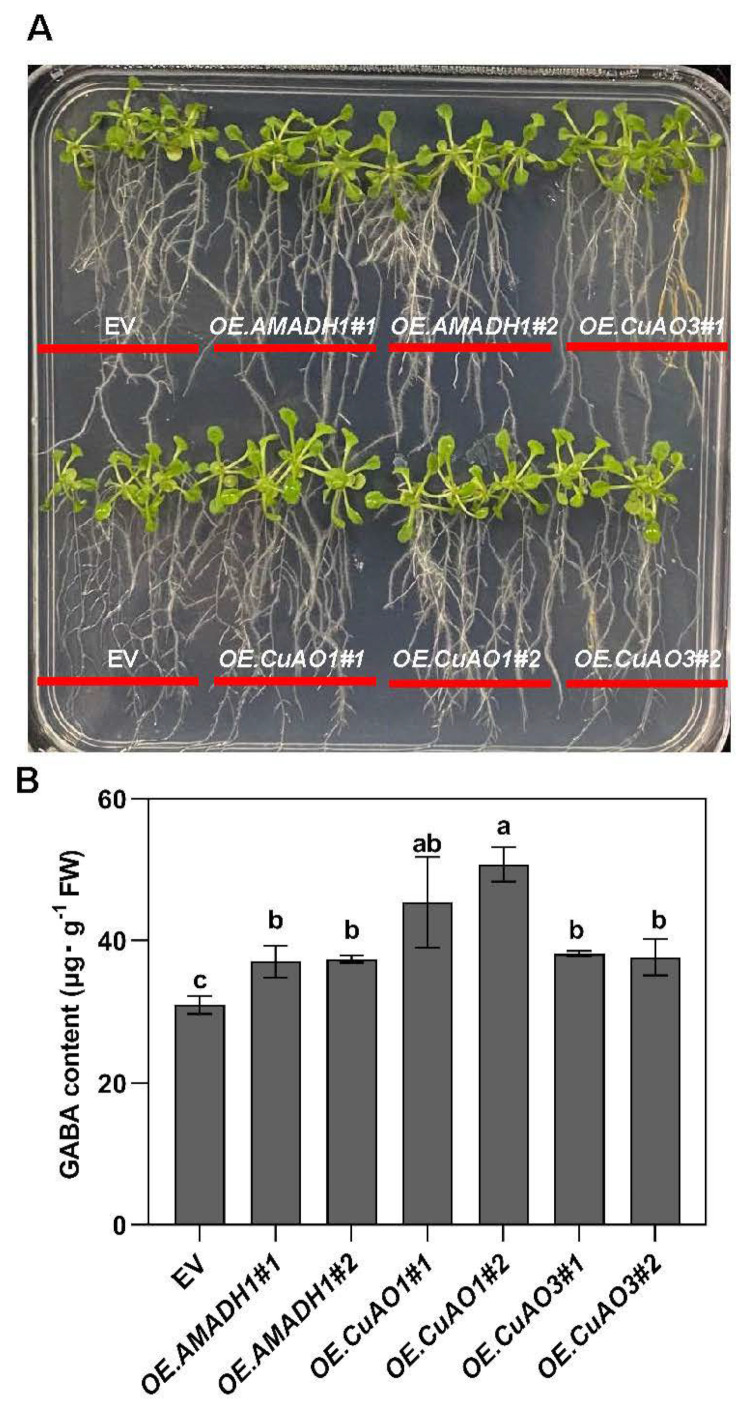
Influence of exogenous putrescine on GABA accumulation levels in *CsAMADH1-*, *CsCuAO1-*, and *CsCuAO3*-overexpressing *Arabidopsis* lines. (**A**) Phenotypes of 10-day-old seedlings of overexpressing lines and empty vector (EV) on 1/2 MS medium supplied with 0.5 mM putrescine. (**B**) The GABA accumulation level of 10-day-old seedlings of overexpressing lines and EV on 1/2 MS medium supplied with 0.5 mM putrescine. Different lowercase letters over columns indicate significant differences between lines (*p* < 0.05).

The fluctuation in GABA content will affect the content of putrescine, so we tested whether GABA shunt-related genes can promote the accumulation of GABA by adding putrescine externally. After 7 days of growth on ½ MS agar medium supplemented with 0.5 mM putrescine (Figure 4A), the GABA content of *CsAMAHD1-*, *CsCuAO1-*, and *CsCuAO3*-overexpression *Arabidopsis* lines was significantly higher than that of empty vector control plants (Figure 4B). Interestingly, the GABA contents of both *CsCuAO1*-overexpressing *Arabidopsis* lines were significantly higher than those of *CsCuAO3*-overexpressing lines (Figure 4B). Therefore, *CsAMAHD1-* and *CsCuAO1*-overexpressing *Arabidopsis* lines were selected for the subsequent experiments.

### 2.5. The Suppression of Putrescine-Derived GABA-Responsive Genes Reduces the Drought Tolerance of Tea Plants

**Figure 5:**
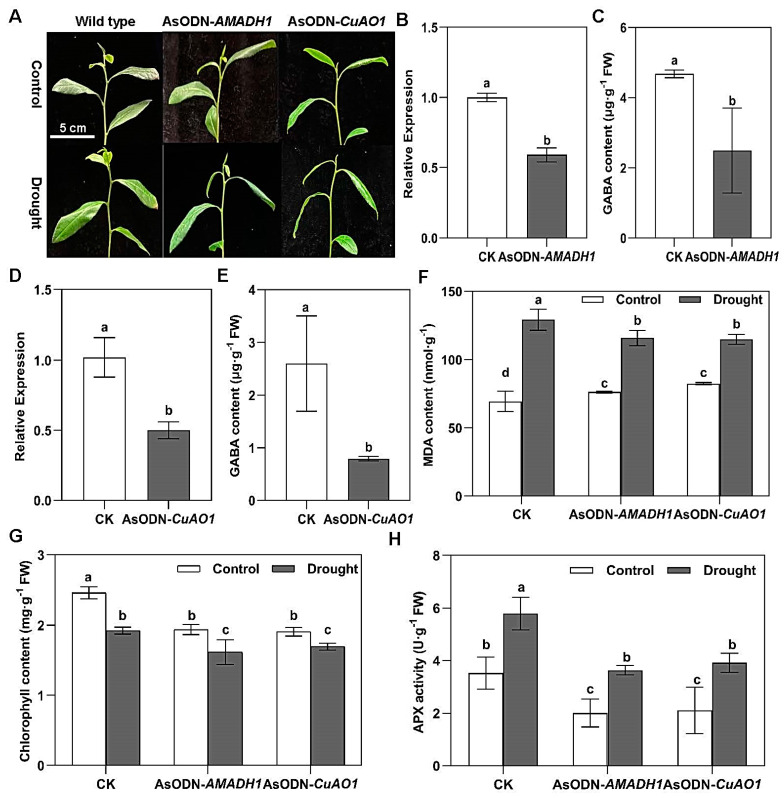
Suppression of putrescine-derived GABA-responsive genes reduces drought tolerance of tea plants. (**A**) Phenotypes of *CsAMADH1*-silenced (AsODN-*AMADH1*), *CsCuAO1*-silenced (AsODN-*CuAO1*), and wild-type tea plants under control or 24 h of drought stress. Scale bar = 5 cm. (**B**) Relative expression level of *CsAMADH1* in *CsAMADH1*-silenced (AsODN-*AMADH1*) and wild-type (CK) tea plants under normal conditions. (**C**) The GABA concentration in *CsAMADH1*-silenced (AsODN-*AMADH1*) and wild-type (CK) tea plants under normal conditions. (**D**) Relative expression level of *CsCuAO1* in *CsCuAO1*-silenced (AsODN-*CuAO1*) and wild-type (CK) tea plants under normal conditions. (**E**) The GABA concentration in *CsCuAO1*-silenced (AsODN-*CuAO1*) and wild-type (CK) tea plants under normal conditions. (**F**) The MDA content in *CsAMADH1*-silenced (AsODN-*AMADH1*), *CsCuAO1*-silenced (AsODN-*CuAO1*), and wild-type (CK) tea plants under control or 24 h of drought stress. (**G**) Chlorophyll content in *CsAMADH1*-silenced (AsODN-*AMADH1*), *CsCuAO1*-silenced (AsODN-*CuAO1*), and wild-type (CK) tea plants under control or 24 h of drought stress. Different lowercase letters over columns indicate significant differences between treatments (*p* < 0.05). (**H**) Apx activity in CsAMADH1-silenced (AsODN-AMADH1), CsCuAO1-silenced (AsODN-CuAO1), and wild-type (CK) tea plants under control or 24 h of drought stress. Different lowercase letters over columns indicate significant differences between treatments (*p* < 0.05).

After 24 h of drought, *AMADH1*- and *CuAO1*-knockdown tea plants showed more obvious wilt symptoms than controls (Figure 5A). When the expression of *CsAMAH1* and *CsCuAO1* was knocked down after incubation for 24 h (Figure 5B,D), the corresponding GABA contents were decreased two times (Figure 5C,E). The MDA contents of the *AMADH1*- and *CuAO1*-knockdown tea plants were two times higher than that of the control group. In the meantime, the MDA content in the knockdown plants increased significantly after 24 h of drought treatment (Figure 5F). Moreover, the chlorophyll contents of the knockdown tea plants were reduced significantly under normal treatment. Interestingly, after 24 h of drought treatment, the chlorophyll content of the knockdown plants was significantly lower than that of the CK (Figure 5G). This was also reflected in the APX activity (Figure 5H), which was always lower in the knockdown plants than in the CK, under both normal and drought treatments. Taken together, our results provided evidence that either *CsCuAO1* or *CsAMADH1* conferred drought tolerance by modulating GABA levels in tea plants.

### 2.6. Overexpressing Arabidopsis Lines Exhibit High Tolerance to Drought

**Figure 6:**
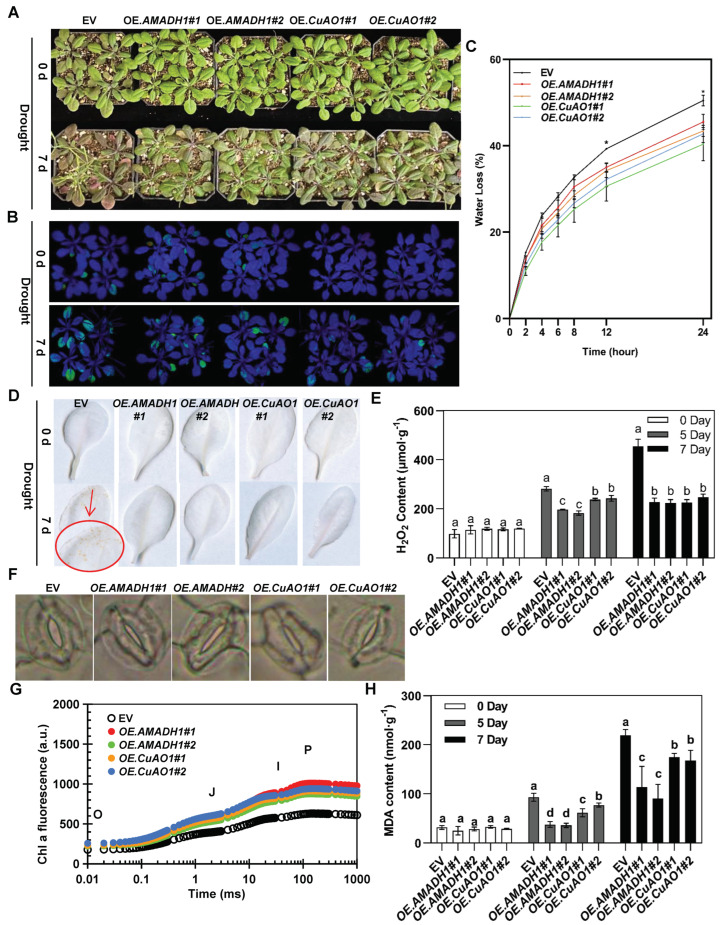
Overexpressing *Arabidopsis* lines exhibit high tolerance during drought treatment. (**A**) Phenotypes of two dependent lines of *CsAMADH1*-overexpressing and *CsCuAO1*-overexpressing 4-week-old *Arabidopsis* before and after 7 d of drought treatment. The overexpressing empty vector (EV) was set as control. (**B**) In vivo chlorophyll fluorescence phenotypes of *Arabidopsis* lines before and after drought treatment. (**C**) Relative water loss of *Arabidopsis* lines during drought treatment. Asterisks show significant differences between the overexpressing lines and EV (*, *p* < 0.01). (**D**) The DAB staining of *Arabidopsis* lines before and after drought treatment. (**E**) The H_2_O_2_ content in leaves of *Arabidopsis* lines after 7 d of drought treatment. Different lowercase letters over columns indicate significant differences between lines (*p* < 0.05). (**F**) Stomatal images in guard cells of *Arabidopsis* leaves after 7 d of drought treatment. Scale bar = 10 μm. (**G**) Chlorophyll fluorescence transients and OJIP test after 7 d of drought treatment. (**H**) The MDA content of *Arabidopsis* leaves after 7 d of drought treatment. Different lowercase letters over columns indicate significant differences between lines (*p* < 0.05).

After 7 days of drought treatment, the *CsAMADH1*- and *CsCuAO1*-overexpressing *Arabidopsis* lines showed reduced phenotypic damage relative to the EV under drought treatment (Figure 6A,B). At 3 and 5 days of drought treatment, transgenic *Arabidopsis* also showed less damage than the EV (Figure S2A). The soil water content was monitored to indicate drought stress in *Arabidopsis* (Figure S2B). The fresh weight of the detached leaves was measured over 24 h to determine the relative water loss of each *Arabidopsis* line, and relative water loss was slower for the *CsAMADH1*- and *CsCuAO1*-overexpressing *Arabidopsis* lines than in the EV transgenic plants. At the end of dehydration, the levels of water loss for the transgenic lines were calculated as 45.48% (*OEAMADH 1#1*), 43.41% (*OEAMADH1#2*), 40.36% (*OECuAO1#1*), 42.75% (*OECuAO1#2*), and 50.49% (EV) (Figure 6C).

The *CsAMADH1*- and *CsCuAO1*-overexpressing *Arabidopsis* lines showed fewer small yellow spots (Figure 6D), which indicated less H_2_O_2_ accumulation (Figure 6E). On day 7, H_2_O_2_ accumulation in the EV was two times higher than that in the overexpression lines. Microscope observations showed that the stomatal aperture of the *CsAMADH1*- and *CsCuAO1*-overexpressing *Arabidopsis* lines was smaller after 7 days of drought treatment (Figure 6F and Figure S2C), which is consistent with the corresponding relative water loss data.

After 7 days of drought treatment, changes in chlorophyll and fluorescence in the *CsAMADH1*- and *CsCuAO1*-overexpressing lines differed from those in the EV (Figure 6G). The fluorescence levels at OJIP of the *CsAMADH1*- and *CsCuAO1*-overexpressing *Arabidopsis* lines were significantly higher than those of the EV. The results showed that the *CsAMADH1*- and *CsCuAO1*-overexpressing lines significantly increased the OJIP curve compared with the EV under drought treatment. This was also consistent with the net photosynthetic rate and stomatal conductance of *Arabidopsis* leaves (Figure S3A,B).

The MDA content of *Arabidopsis* leaves increased with the drought treatment time, but the *CsAMADH1*- and *CsCuAO1*-overexpressing *Arabidopsis* lines had lower MDA contents following exposure to drought treatment than the EV control plants (Figure 6H). With increasing drought time (0, 5, and 7 days), the chlorophyll content of *Arabidopsis* leaves decreased, but the chlorophyll content was always higher in the overexpressing lines than the EV controls (Figure S3C). Taken together, under drought treatment, *CsAMADH1* and *CsCuAO1* played a positive role in photosynthetic performance and reduced water loss by adjusting the stomatal aperture in response to drought stress.

### 2.7. Transient Assay and Transgenic Analysis of CsCuAO1-CsAMADH1 Co-Expression

**Figure 7:**
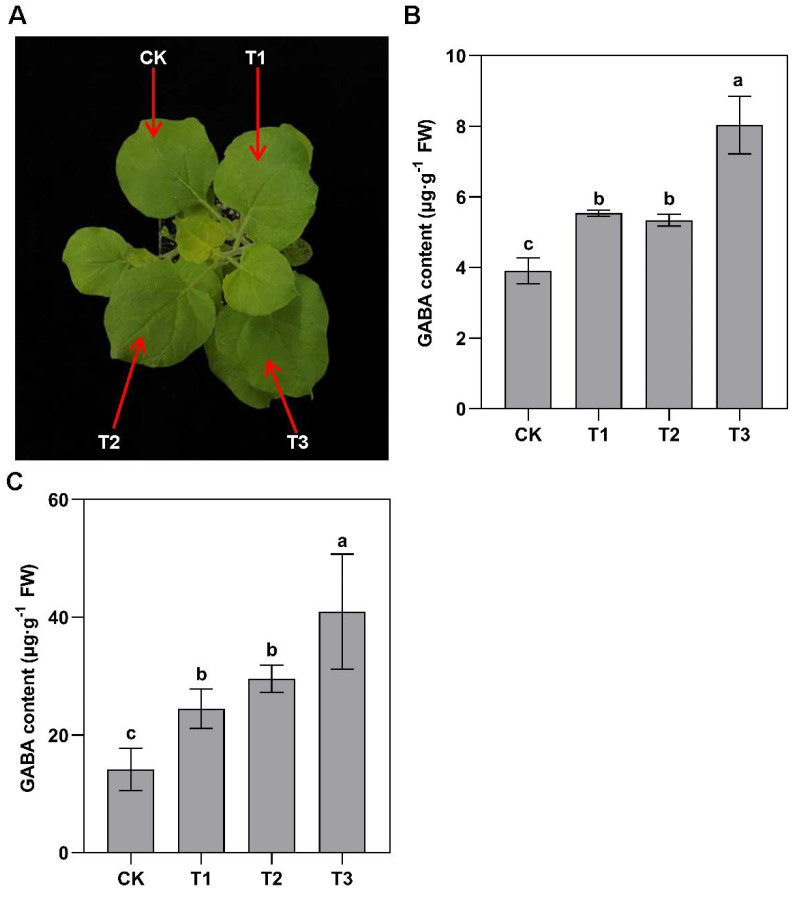
Transient assay and transgenic analysis of co-expression of CsCuAO1–CsAMADH1. (**A**) Phenotypes of *N. benthamiana* leaves following *Agrobacterium* infiltration harboring the respective plasmids after 1 day. CK: EV was injected; T1: CsAMADH1 was injected; T2: CsCuAO1 was injected; T3: CsAMADH1 and CsCuAO1 were injected together. (**B**) The GABA accumulated in leaves at 3 days after *Agrobacterium* infiltration. (**C**) The GABA accumulation level of 10-day-old seedlings of overexpressing, co-expression, and EV lines on 1/2 MS medium supplied with 0.5 mM putrescine. Different lowercase letters over columns indicate significant differences between lines (*p* < 0.05).

After 3 days of incubation, the GABA content of *N. benthamiana* leaves in T1 and T2 was significantly higher than that of the CK. Moreover, the GABA content of T3 increased much more compared with T1 and T2 (Figure 7A,B).

After separately supplying putrescine to the overexpressing pBI121–GFP EV (CK), CsAMADH1 (T1), CsCuAO1 (T2), and CsCuAO1–CsAMADH1 (T3) Arabidopsis lines (Figure S4A,B) for 3 days, the GABA contents of T1 and T2 significantly rose (Figure 7C); however, the GABA content was higher for T3 than for T1 or T2. Overall, these results suggested that CsAMADH1 that is associated with CsCuAO1 played an important role in GABA accumulation.

## 3. Discussion

### 3.1. CsCuAO1 and CsAMADH1 Encode Functional Proteins

Very recently, we reported that *CuAO1* and *AMADH1* were involved in the regulation of GABA accumulation in tea plants through the polyamine degradation pathway [20]. The accumulation of GABA is induced by various stresses, and understanding of *CuAO1* and *AMADH1* is critical to elucidate their physiological function in response to drought stress. The *CuAO* plays an important role in ROS scavenging, which can regulate many development processes and defense responses [22,24]. Plant AMADHs have been reported to perform substrate-dependent NADH production, which could exhibit antioxidant activity [26]. In this study, the *Agrobacterium*-mediated transient expression assay suggested that *CsAMADH1* worked coordinately with *CsCuAO1* in participating in the putrescine-derived GABA accumulation pathway (Figure 7A,B). In addition, the GABA content of *CsCuAO1–CsAMADH1* co-overexpressing *Arabidopsis* was higher than individual *CsAMADH1*- or *CsCuAO1*-overexpressing *Arabidopsis* plants (Figure 7C). Moreover, the *CsAMADH1*- and *CsCuAO1*-overexpressing lines showed less water loss, less H_2_O_2_ production, and better performance of stomatal movement and photosynthesis than the EV control plants (Figure 6). The above data indicated that the *CuAO1* and *AMADH1* genes isolated from *C. sinensis* encoded functional proteins.

### 3.2. GABA Contributes to Drought Stress Tolerance through Affecting ROS Scavenging and Stomatal Closure in Tea Plants

Drought affects plant growth and crop production, and the GABA regulation of the drought response has been extensively studied [4,8,9,11]. The ROS are controlled by the antioxidant defense system, and ROS accumulation can damage plant cells and photosystem II [30]. Histochemical staining with DAB showed that overexpressing lines accumulated less ROS under drought (Figure 6D,E), which is consistent with the lower MDA level (Figure 6H). Using GABA spraying and a gene suppression system, we demonstrated that the increased GABA level improved antioxidant activity (APX and MDA), while a reduction in GABA inhibited the antioxidant defense system, which was closely related to ROS scavenging activity (Figure 1 and Figure 5). Furthermore, our data indicated that the lower accumulation of ROS in overexpressing lines may have contributed to the higher putrescine contents, which are reported to play a positive role in the alleviation of ROS injury [31]. Photosynthetic capacity is a major leaf trait, which is reflected in the maximum photosynthetic rate and response to environmental stresses [32]. Interestingly, due to less water loss, the overexpressing lines had better photosynthetic capacity compared with the EV control plants under soil drought conditions (Figure 6G and Figure S3A,B).

Stomatal movement is closely correlated with plant water loss, which affects plant growth under drought conditions; the narrower the stomatal aperture, the lower the water loss [33]. Recently, GABA was reported as a novel stomatal behavior modifier, which fine-tunes the gas exchange and intrinsic water use efficiency in multiple species [11]. Stomatal closure is induced by rapidly synthesized GABA through downstream signaling pathways, which causes a decrease in CO_2_ uptake, as well as a reduction in photosynthetic efficiency and transpiration (Figure 5) [10,11]. In the present study, we showed that GABA, accumulated by the overexpression of *CsCuAO1* and *CsAMADH1*, played an important role by altering stomatal closure under drought stress (Figure 6). Our data are consistent with GABA playing a crucial role in modulating stomatal movement, which also further suggested that GABA may inhibit stomatal opening and contribute to drought tolerance.

### 3.3. GAT1 Acts as a GABA Transporter in Tea Plants

**Figure 8:**
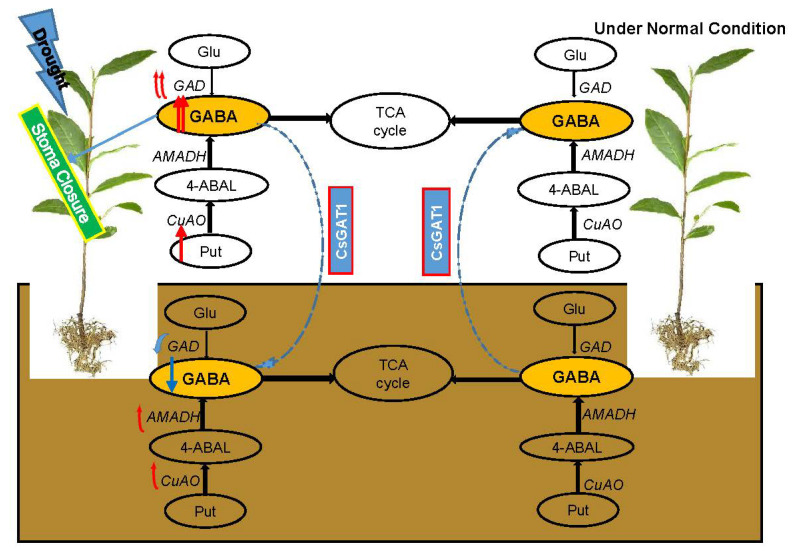
Tentative working model for *CsCuAO1* associated with *CsAMADH1* participating in the modulation of GABA levels in tea plants under drought stress. When tea plants were grown under normal conditions, some GABA was transported from underground to leaves, which regulated CsGAT1 activity. When tea plants were affected by drought stress, due to the upregulation of GAD activity and putrescine, GABA was greatly accumulated and balanced by CsGAT1, which functioned in ROS scavenging and stomatal closure.

In the cytosol, GABA is synthesized from glutamate via GAD activity and the putrescine degradation pathway through CuAO and AMADH, which were identified in this study. The GABA is then transported into mitochondria by GAT or GABA permease (GABP). In *Arabidopsis*, AtGAT1 has been shown to be a specific GABA transporter protein [29]. The AtGABP-mediated movement of GABA from the cytosol into mitochondria is important for energy metabolism [34]. In *Arabidopsis*, AtALMT1 has also been shown to transport GABA [35]. In this study, we characterized a GABA transporter, CsGAT1, in tea plants (Figure 3C). Furthermore, yeast complementation assays indicated that CsGAT1 was functional for transporting GABA, but not other amino acids (Figure S1). The yeast mutant could only grow normally on YNB medium with arginine as the sole nitrogen source but could not grow if other amino acids were the sole nitrogen source [36]. A further interesting feature of *CsGAT1* was that it showed a very high correlation with the GABA accumulation level in different organs (Figure 7B). When tea plants were grown under normal conditions, some GABA was transported from the underground to leaves, which regulated the activity of CsGAT1. When tea plants were affected by drought stress, due to the upregulation of GAD activity and putrescine, GABA was significantly accumulated and balanced by *CsGAT1*, which functioned in ROS scavenging and stomatal closure (Figure 8).

We further elucidate the role of GABA in plant drought tolerance, prove that *CsAMAHD1* and *CsCuAOs* cooperate to promote the accumulation of GABA in roots to improve plant drought tolerance and that GABA homeostasis in different parts of plant under drought stress is mediated by the transport enzyme CsGAT1, and clarify the accumulation and circulation mechanism of GABA in plants under drought stress.

## 4. Materials and Methods

### 4.1. Plant Materials and Growth Conditions

Tea plants (*Camellia sinensis* var. zhongcha108), wild-type *Nicotiana benthamiana*, and *Arabidopsis* (*Arabidopsis thaliana*, accession Col-0) were used in this project. CsAMAHD1-, CsCuAO1-, and CsCuAO3-overexpressing Arabidopsis lines were generated as follows: The respective coding sequences (CDSs) of CsAMADH1, CsCuAO1, and CsCuAO3 (Table S1) were cloned into the XbaI and BamHI sites of the pBI121–GFP vector and incorporated into Agrobacterium tumefaciens GV3101 strain, and then transformed into Arabidopsis plants through Agrobacterium-mediated transformation by the floral dip method [37]. The pBI121–GFP empty vector (EV) was also transferred into Arabidopsis as the control. The specific primer pairs used are listed in Table S2.

Tea plants were grown in an artificial climate incubator with 16 h of light (25 °C)/8 h of dark photocycle (20 °C). The *N. benthamiana* plants were grown in soil at 26 °C with 16 h of light/8 h of dark photocycle. For Arabidopsis plants, seeds were surface-sterilized in 75% ethanol for 5 min and grown on 1/2 MS agar medium containing 1% sucrose at 22 °C with 16 h of light/8 h of dark photocycle. Arabidopsis plants were also grown in soil in an artificial climate incubator at the same light regime at 22 °C.

### 4.2. Drought Stress Treatments

For tea plants, 20% (*w*/*v*) polyethylene glycol (PEG) 6000 was used for drought treatments [38]. Seedlings were grown in 200 mL of nutrient solution (control) and treated with 200 mL of 20% PEG for 24 h as drought treatment. For the Mock samples, plants were sprayed with ddH_2_O. For GABA-treated samples, plants were sprayed with an equal amount of 5 mM GABA. Each treatment had three independent biological replicates, each including three randomly selected tea plants. Plant samples were collected, consisting of a bud and two leaves, immediately frozen in liquid nitrogen, and stored at −80 °C for further analysis.

Transgenic *Arabidopsis* plants after 30 days of growth (10 days on 1/2 MS agar medium and 20 days in soil) were selected for drought treatment, which consisted of watering with 2 L of water on the 10th day, and 1 L on the 20th day. Drought stress treatment began on the 30th day (0 day). The collected plant samples were immediately frozen in liquid nitrogen and stored at −80 °C for further analysis.

### 4.3. Gene Expression Analysis

Isolation of total RNA from plant samples was performed using a FastPure Universal Plant Total RNA Isolation Kit (Vazyme Biotech Co., Ltd., Nanjing, China) according to the manufacturer’s instructions. Then, reverse transcription of cDNA from total RNA was performed using HiScript II Q RT SuperMix (Vazyme Biotech Co., Ltd.). Quantitative real-time (qRT) PCR assays were performed in a Bio-Rad CFX96 machine (Bio-Rad, Hercules, CA, USA). The reagent used for the qRT-PCR experiment was ChamQ Universal SYBR qPCR Master Mix (Vazyme Biotech Co., Ltd.). The relative gene expressions were calculated using the 2^−ΔΔCT^ method (*Csβ-actin* gene was the internal control). Each sample was replicated at least 3 times. The primer pairs used in this study are listed in Table S2.

### 4.4. Functional Verification of Transgenic Arabidopsis Plants

Transgenic *Arabidopsis* plants overexpressing *CsAMADH1*, *CsCuAO1*, and *CsCuAO3* were sown on 1/2 MS agar medium containing kanamycin. *Arabidopsis* containing pBI121–GFP vector only was used as the control. When the *Arabidopsis* seedlings reached the four-true-leaves stage, they were transplanted onto a 1/2 MS agar medium containing 0.5 mM putrescine on an ultra-clean bench. After 7 days, the *Arabidopsis* leaves were collected to determine GABA content. Each group was replicated at least 3 times.

### 4.5. Gene silencing of CsAMADH1 and CsCuAO1 in Tea Plants

Candidate three antisense oligodeoxynucleotides (AsODNs) that were located in the domain were selected using Soligo software V1.0 with *CsAMADH1* and *CsCuAO1* as the input sequences (Table S1). Three primers were diluted separately in 20 μM and mixed. To silence expression of *CsAMADH1* and *CsCuAO1* in tea leaves, 1 mL of 20 μM AsODN-CsAMADH1 solution and 1 mL of 20 μM AsODN-CsCuAO1 solution were separately injected into different tea seedlings, and seedlings injected with deionized water were used as the CK [4]. Samples were collected and stored at −80 °C after 24 h of incubation. RNA extraction and qRT-PCR were performed to verify the expression of target genes. If the expression level is down-regulated by more than 40%, the silencing is considered successful, and those samples are used for further detection. Each group had at least 5 tea seedlings for injection.

### 4.6. Determination of GABA Contents

A total of 0.2 g of sample was ground in liquid nitrogen and placed into a 10 mL centrifuge tube, and 2 mL of 0.02 mM HCl was added. After 8 h of incubation at 4 °C, the samples were centrifuged at 4 °C and 12,000 rpm for 15 min, and the supernatant was transferred into a new centrifuge tube. An equal volume of 4% sulfosalicylic acid was added and mixed. The mixture was then filtered through a 0.22-μM organic membrane, and GABA or amino acid content was determined using an amino acid composition analyzer (Hitachi L-8900; Hitachi, Osaka, Japan). The GABA contents were obtained by calculating the peak area and comparing it to the standard solution (0.1 μmol GABA) [20]. Each sample was replicated at least 3 times.

### 4.7. Putrescine Content Determination

The putrescine content in samples was determined by high-performance liquid chromatography (Waters ACQUITY HPLC) [39]. Samples were homogenized with 5% pre-cooling perchloric acid. After centrifugation at 12,000× *g* for 20 min at 4 °C, the supernatant was mixed with 2 M NaOH and benzoyl chloride and then incubated at 37 °C for 30 min. Then, the sample was completely mixed with ether. The mixture was centrifuged at 3000× *g* at 4 °C for 10 min for phase separation. After evaporation of the organic phase, the sample was dissolved in 0.5 mL of methanol and detected by HPLC (C_18_ column, 15 cm × 0.39 cm × 4 μm) at 254 nm. One detection cycle is consisting of 60 min at a flow rate of 1.0 mL min^−1^ at 30 °C; i.e., 42% acetonitrile for 25 min, followed by being increased up to 100% acetonitrile during 3 min, 100% acetonitrile for 20 min, decreased down to 42% acetonitrile during 3 min, and then 42% acetonitrile for 9 min. Each sample was replicated at least 3 times.

### 4.8. Determining Electric Conductivity

A total of 1 g of sample was cut into 1 cm segments and washed with deionized water three times. The segments were put into a 15 mL centrifuge tube with 10 mL of deionized water. The segment was blown with vacuum pump for 15 min. After standing for 20 min, the electric conductivity of the solution was measured using a DDS-307 conductivity meter (Shanghai INESA Scientific Instrument Co., Ltd., Shanghai, China) with deionized water as control. The tubes were placed into a 100 °C water bath for 15 min and cooled for 15 min, and then, electric conductivity was measured again. The ratio of the two measurements was the electric conductivity of the sample. Each sample was replicated at least 3 times.

### 4.9. Determination of MDA and Chlorophyll Contents 

The MDA content from plant samples was measured using Micro MDA Assay Kit (Beijing Solarbio Science & Technology Co., Ltd., Beijing, China) according to the manufacturer’s instructions. Each sample was replicated at least 3 times.

A total of 0.1 g of sample was ground with a small amount of quartz sand, calcium carbonate powder, and 2.5 mL of anhydrous ethanol and then transferred into a 10 mL centrifuge tube, and 7.5 mL of anhydrous ethanol was added. After centrifugation at 4 °C and 5000× *g* for 10 min, 200 μL of the supernatant was applied to determine absorbance at 665 and 645 nm. The chlorophyll content of the sample was calculated according to the simplified formula: chlorophyll content (mg·g^−1^, FW) = 0.663 A665 + 0.808 A649. Each sample was replicated at least 3 times.

### 4.10. Measurement of Stomatal Aperture

For tea plants, stomata were observed by imprinting method. Briefly, nail polish was applied evenly to the lower epidermis of tea leaves. After drying, the nail polish sheet was torn, and deionized water was added to it to make glass slides. For *Arabidopsis*, the lower epidermis of leaves was quickly torn and added with anhydrous ethanol to make glass slides. Then, stomata were photographed using an upright fluorescence microscope (Leica LMD7000; Leica, Wetzlar, Germany). Each treatment included at least three biological replicates, and ImageJ software V 1.0 was used to measure aperture of more than 20 stomatal cells in each sample.

### 4.11. Determination of Photosynthetic Parameters and Chlorophyll Fluorescence Parameters

The photosynthesis-related parameters of samples were determined using a Portable Photosynthesis Measurement System Li-6400 (Beijing Ecotek Technology Co., Ltd., Beijing, China). Each treatment was measured at least five times at 11 am on the same day, and the built-in light intensity was set to 600 μmol·m^−2^·s^−1^.

The in vivo chlorophyll fluorescence phenotypes of *Arabidopsis* plants were determined and photographed using a Modulated Fluorescence Imaging System (Heinz Walz GmbH, Nuremberg, Germany) and Imaging Win software V 2.0 after 30 min of dark acclimation. Each treatment with three repeats was measured at the same time.

Leaves were dark-acclimated for 30 min and clamped by a leaf clip. Chlorophyll fluorescence rise kinetics were determined at room temperature in darkness using a Handy-PEA fluorometer (Plant Efficiency Analyzer, Hansatech Instruments Ltd., King’s Lynn, Norfolk, UK) [40,41]. All samples were replicated at least 15 times.

### 4.12. Detection of Oxidative Damage in Plants

The H_2_O_2_ content from plant samples was measured using a Hydrogen Peroxide Assay Kit (Beijing Solarbio Science & Technology Co., Ltd.) according to the manufacturer’s instructions. 

For visualization of H_2_O_2_ generation as a result of drought stress, the DAB method was used for tissue staining. Leaves of *Arabidopsis* samples were placed in 1 mg·mL^−1^ DAB solution (pH 3.8, 50 mM·L^−1^ Tris-HCl) and stained for 8 h in darkness. According to the ratio of anhydrous ethanol:acetic acid:glycerol = 3:1:1 configuration of the proportion of decolorization solution, leaves were placed in a 100 °C water bath until they were completely decolorized [42].

The APX activity from plant samples was determined using an Ascorbate APX Activity Assay Kit (Beijing Solarbio Science & Technology Co., Ltd.). Each sample was replicated at least 3 times.

### 4.13. Determination of Soil Water Content and Free Water Loss

A total of 3 g of *Arabidopsis* rhizosphere soil was placed in a dry aluminum box and dried to constant weight in a 115 °C oven to calculate moisture content.

Transgenic *Arabidopsis* plants grown for 30 days were used for determining water loss. The samples were placed in the same indoor environment, and the sample weight determined after 0, 2,4, 6, 8, 12, and 24 h. Each sample was replicated at least 3 times.

### 4.14. Transient Assay in N. benthamiana Leaves

The *Agrobacterium* separately containing *CsAMADH1*, *CsCuAO1*, and pBI121–GFP plasmids was adjusted to OD600 of 0.6–1.0 by activation buffer solution and then injected into *N. benthamiana* leaves by *Agrobacterium* infiltration. The leaves injected with pBI121–GFP plasmids were used as control (CK). After the dark treatment for 12 h, samples were taken after 60 h of incubation to quantify the GABA content [43]. Each sample was replicated at least 3 times.

### 4.15. Pollen Hybridization and Functional Verification of Transgenic Arabidopsis

The *CuAO1-* and *AMADH1*-overexpressing transgenic *Arabidopsis* were used as male and female parents, respectively, for hybridization. First, all stamens from *AMADH1*-overexpressing *Arabidopsis* were cut and only the stigma retained, and this was smeared with the stamens of *CuAO1*-overexpressing *Arabidopsis*. The stigma was then covered with a small bag and kept wet. Only the fruit clip growing from this stigma was retained. After sowing the harvested seeds, only the *Arabidopsis* plants with two gene bands of *CsAMADH1* and *CsCuAO1* after PCR verification were retained. Hybrid *Arabidopsis* was transferred to the media supplied with 5.0 mM putrescine. Samples were taken after 3 days to determine GABA content.

### 4.16. Phylogenetic Tree Construction

The ALMT, GABP, and GAT protein sequences of *Arabidopsis* were derived from the plant transcription factor database, PlantTFDB (http://planttfdb.cbi.pku.edu.cn/, accessed on 11 November 2022). The HMMER software V3.0 was employed for specific domain searching from the tea plant genome database (http://tpia.teaplants.cn/, accessed on 11 November 2022) with the default parameter E-value < 1 × 10^−5^. MEGA7.0 software, with default parameters, was used to construct a neighbor–junction phylogenetic tree.

### 4.17. Yeast Spot Experiment

The CDS of the *CsGAT1* was cloned into the pYES2 vector. Yeast transformation kit (Zymo Research, Irvine, CA, USA) was used to transform the yeast strain 22Δ10α mutant (23344c background) with pYES2-CsGAT1 or the EV pYES2. The transformants were selected on yeast YNB (galactose) solid medium lacking uracil and supplemented with 1 mM (NH_4_)_2_SO_4_ [44].

Single colonies were picked and cultured in 20 mL of YNB (galactose) liquid medium at 30 °C and 200 rpm/min and shaken until OD600 was 0.6–0.8 (about 48 h). Of yeast solution, 4 mL was collected and centrifuged at 5000 rpm for 2 min. The supernatant was removed (aseptic operation), and the yeast was resuspended with sterile water. The yeast solution was diluted with sterile water to OD600 of 0.6 (the difference in OD value between each sample did not exceed 0.02). A total of 1.5 μL of yeast solution was inoculated on YNB (galactose) solid medium with different amino acids as the sole nitrogen source. The growth of yeast plaque was observed and photographed after incubating at 30 °C for 3 days.

### 4.18. Statistical Analysis

At least three biological replicates were used in each of the aforementioned tests. SPSS v20.0 (IBM, New York, NY, USA) was used to perform statistical analyses. Significant differences were confirmed with ANOVA and Duncan’s test and least significant difference (LSD) tests at the 0.05 probability level. A *p*-value of <0.05 was considered statistically significant. 

## Data Availability

All data are available in the manuscript or the Supplementary Materials.

## Supplementary Materials

The supporting information can be downloaded at https://www.mdpi.com/article/10.3390/ijms25020992/s1.
