# Supplementary material for: CsCuAO1 Associated with CsAMADH1 Confers Drought Tolerance by Modulating GABA Levels in Tea Plants
Source: Int J Mol Sci. 2024 Jan 12;25(2):992. doi: 10.3390/ijms25020992 (PMC10815580; doi:10.3390/ijms25020992)
Supplement: Supplementary file 1 [file ijms-25-00992-s001.zip › ijms-2760062-supplementary.pdf]

International Journal of *Molecular Sciences*

MDPI

Article

CsCuAO1 Associated with CsAMADH1 Confers Drought Tolerance by Modulating GABA Levels in Tea Plants

Yu Cao [1,†], Yiwen Chen [1,†], Nuo Cheng [1], Kexin Zhang [1], Yu Duan [1], Shimao Fang [1,2], Qiang Shen [2], Xiaowei Yang [2], Wanping Fang [1] and Xujun Zhu [1,*]

[1] College of Horticulture, Nanjing Agricultural University, Nanjing 210095, China; Yu Cao, 2020104087@stu.njau.edu.cn; Yiwen Chen, 2021104084@stu.njau.edu.cn; Nuo Cheng, 14221125@stu.njau.edu.cn; Kexin Zhang, 2019104086@njau.edu.cn; Yu Duan, 2018204034@njau.edu.cn; Shimao Fang, fsm12340@163.com; Wanping Fang, fangwp@njau.edu.cn

[2] Tea Research Institute, Guizhou Provincial Academy of Agricultural Sciences, Guiyang 417100, China; Qiang Shen, Shenqiang_GZU@163.com; Xiaowei Yang, yangxiaowei_GZU@163.com

* Correspondence: author: Prof. Xujun Zhu, College of Horticulture, Nanjing Agricultural University, Nanjing 210095, China; Telephone & Fax: +86-25-84395182

† These authors contributed equally to this work.

Supplementary Materials

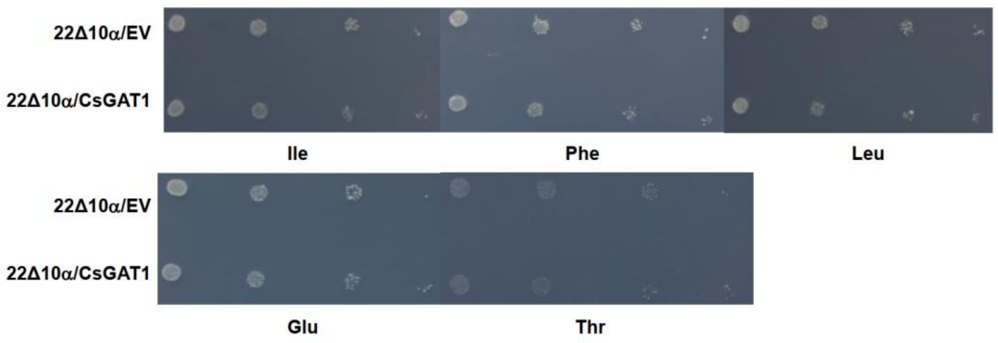

Figure S1. Phenotypes of yeast strains with CsGAT recombinant plasmids grown on solid medium, with amino acid as the only nitrogen source.

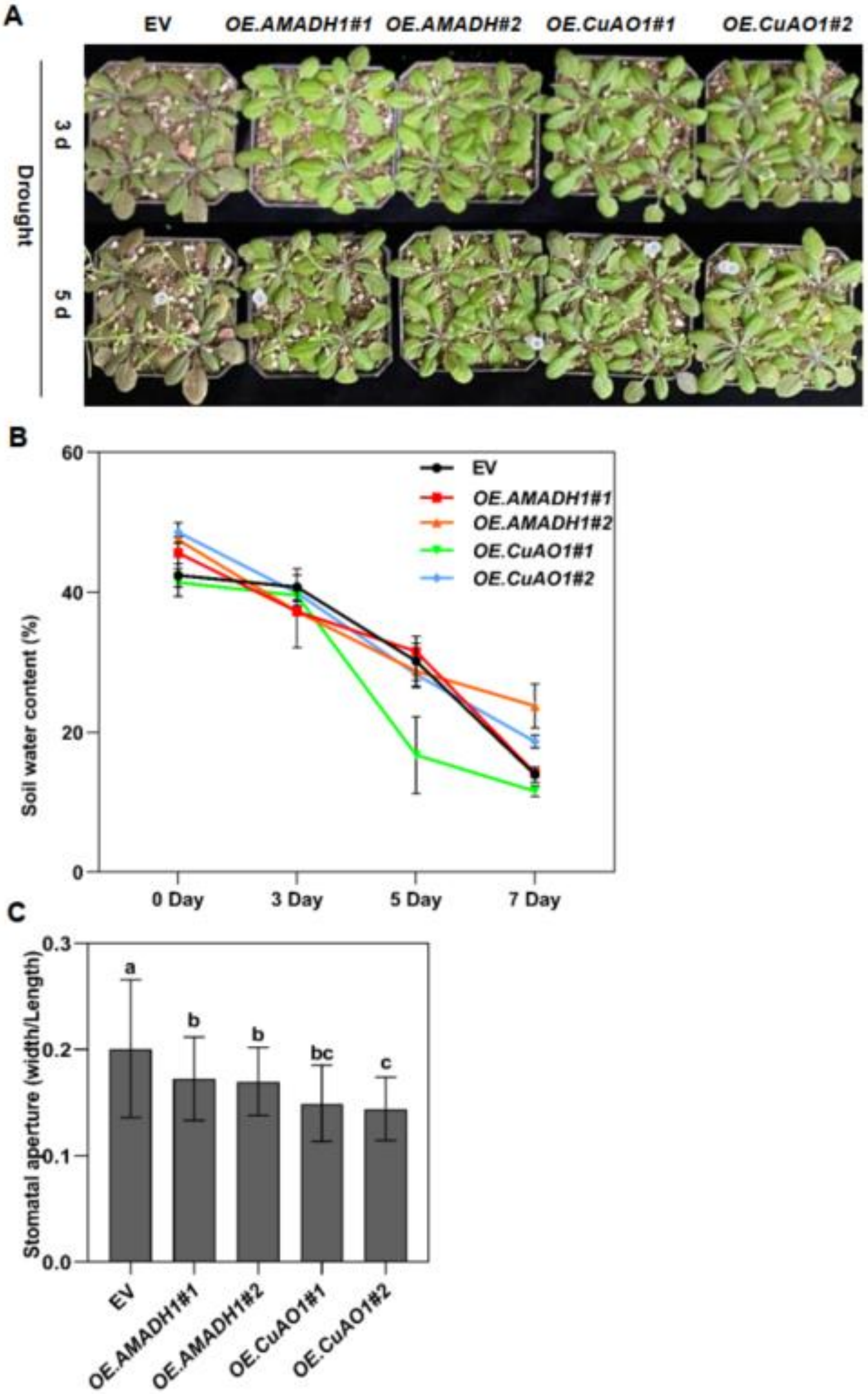

Figure S2. The overexpressing *Arabidopsis* lines exhibited high tolerance during drought treatment. (A) Phenotypes of two dependent lines of *CsAMADH1*-overexpressing and *CsCuAO1*-overexpressing four-week-old *Arabidopsis* before and after 3 and 5 days of drought treatment. The overexpressed empty vector (EV) was set as control. (B) Soil water content for each *Arabidopsis* line during drought treatment. (C) Stomatal apertures in guard cells of *Arabidopsis* leaves after 7 d of drought treatment. Different lowercase letters over columns indicate significant differences between lines ($p < 0.05$).

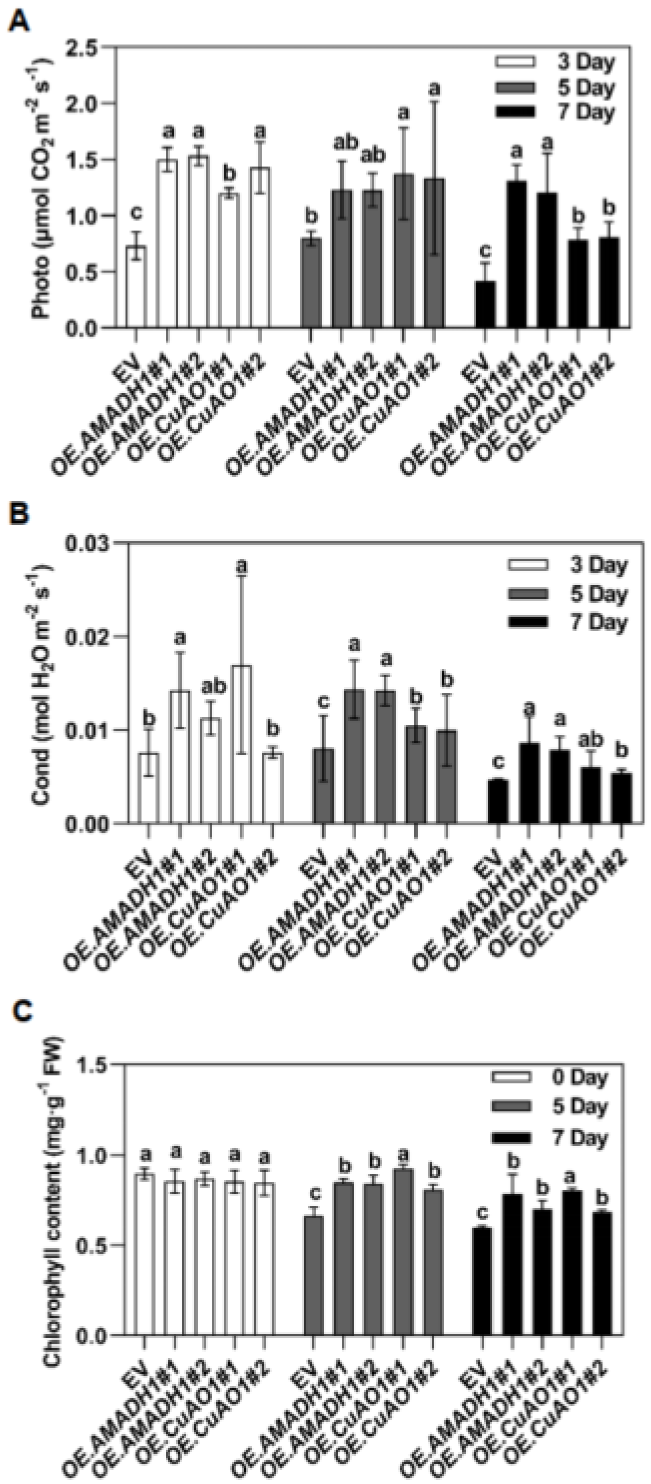

Figure S3. (A) Net photosynthetic rate and (B) stomatal conductance of *Arabidopsis* leaves after 7 d of drought treatment. (C) Chlorophyll content of *Arabidopsis* leaves after 7 d of drought treatment. Different lowercase letters over columns indicate significant differences between lines ($p < 0.05$).

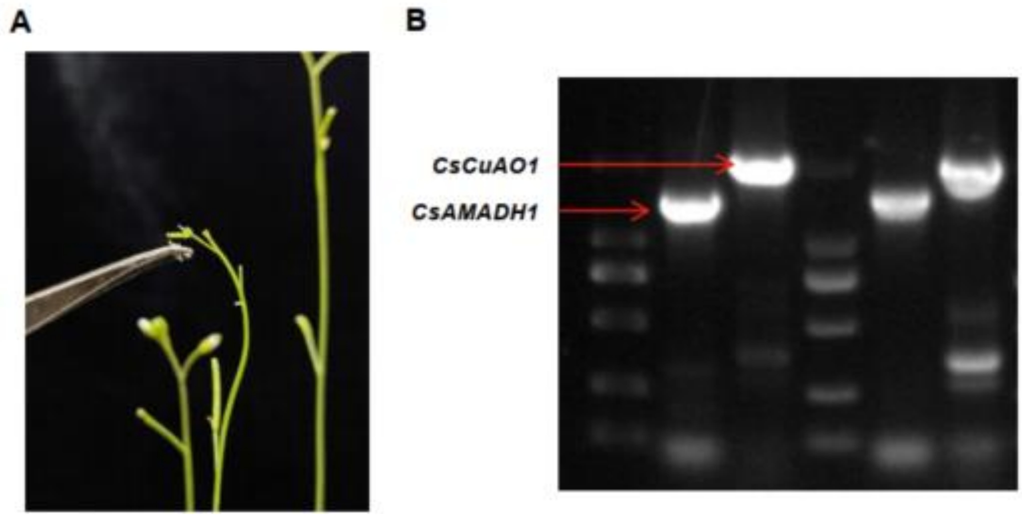

Figure S4. The generation of transgenic co-overexpressing lines of *CsCuAO1–CsAMADH1*. (A) *CuAO1*-overexpressing and *AMADH1*-overexpressing transgenic *Arabidopsis* were used as male and female parents, respectively, for pollen hybridization. (B) Photograph of electrophoresis analysis of co-overexpressing line.

Table S1. Genes used in this study.

Gene name	Gene ID	Template size
CsCuAO1	TEA029860.1	2172bp
CsCuAO3	TEA015253.1	2040bp
CsAMADH1	TEA025182.1	1515bp

Copyright: © 2024 by the authors. Licensee MDPI, Basel, Switzerland. This article is an open access article distributed under the terms and conditions of the Creative Commons Attribution (CC BY) license (https://creativecommons.org/licenses/by/4.0/).

Table S2. Primers used in this study.

Primers	Sequence (5´-3´)	Remarks
Csβ-actin-F	GCCATCTTTGATTGGAATGG	Internal reference
Csβ-actin-R	GGTGCCACAACCTTGATCTT	Internal reference
CsGAT1-F	GCAGTAGTGACAGTGACTT	qPCR
CsGAT1-R	GATGACGACAGACAGTGAT	qPCR
CsGAD1-F	AGTGACATCCAGAAAGTCTTGCT	qPCR
CsGAD1-R	CACCATTAGTCTTCTTCCTACTGAG	qPCR
CsGAD2-F	TTCGACATCTGCAAGGTGCTCCA	qPCR
CsGAD2-R	ACTTGTGTTTTCCTAGCCAAGAC	qPCR
CsGAD3-F	TTTCACATAACAAATGCAACGTC	qPCR
CsGAD3-R	CTCCCTTTGTCTTACCACCCAT	qPCR
CsCuAO1-F	AAACGCCCAACAACAAAT	qPCR
CsCuAO1-R	CGAACAACTCCGAACTAAAC	qPCR
CsCuAO3-F	TATTGAGATTGCTGGACAC	qPCR
CsCuAO3-R	AACCCAAATTCGCCTA	qPCR
CsAMADH1-F	AGCCTGTGAAGAAGAAACG	qPCR

CsAMADH1-R	CCAGCAATAGAACGCAAA	qPCR
CsCuAO1-F	ATGGAAGAAAAGAGCCTCCTC	PCR
CsCuAO1-R	GGCAGAAGCAGAAGCCTT	PCR
CsCuAO3-F	ATGGAAGCTTCAACATTGAG	PCR
CsCuAO3-R	AGGTTTGGCAGTGCAATT	PCR
CsAMADH1-F	ATGGCGATCCCAATACCTACTC	PCR
CsAMADH1-R	AAGCTTTGGCTTTGAAGGAGATGGG	PCR
CsCuAO1-X-F	ACACGGGGGACTCTAGAATGGAAGAAAA-GAGCCTCCTC	PCR
CsCuAO1-B-R	TGACCACCCGGGGATCCGGCAGAAGCAGAAGCCTT	PCR
CsCuAO3-X-F	ACACGGGGGACTCTAGAATGGAAGCTTCAACATT-GAG	PCR
CsCuAO3-B-R	TGACCACCCGGGGATCCAGGTTTGGCAGTGCAATT	PCR
CsAMADH1-X-F	ACACGGGGGACTCTAGAATGGCGATCCCAATAC-CTACTC	PCR
CsAMADH1-B-R	TGACCACCCGGGGATCCAAGCTTTGGCTTT-GAAGGAGATGGG	PCR
AsODN-CsA-MADH1-1	GGCGAGTAGGTATTGGGATC	Gene suppression
AsODN-CsA-MADH1-2	GAGCTGCAGCAGTCATGATC	Gene suppression
AsODN-CsA-MADH1-3	ATGGCTGTCGTTTGCCAGTT	Gene suppression
AsODN-CsCuAO1-1	CATGTGGAGGTGCAGTCGAG	Gene suppression
AsODN-CsCuAO1-2	TTGGGGATTGGTATGTTCCT	Gene suppression
AsODN-CsCuAO1-3	GATTATTTGTGAATGCGCCT	Gene suppression

Disclaimer/Publisher's Note: The statements, opinions and data contained in all publications are solely those of the individual author(s) and contributor(s) and not of MDPI and/or the editor(s). MDPI and/or the editor(s) disclaim responsibility for any injury to people or property resulting from any ideas, methods, instructions or products referred to in the content.
